# Results of applying the 9th edition TNM classification in the staging assessment of nasopharyngeal carcinoma: first data in Vietnam

**DOI:** 10.3389/fonc.2026.1830412

**Published:** 2026-07-15

**Authors:** Dang Nguyen Van, Phan Nguyen Huy, Hoa Le Thi, Viet Hoang Thanh

**Affiliations:** 1 Department of Oncology, Hanoi Medical University, Hanoi, Vietnam; 2 Department of Head and Neck Radiation Oncology, Vietnam National Cancer Hospital, Hanoi, Vietnam

**Keywords:** nasopharyngeal carcinoma, NPC, TNM 9th edition, TNM staging, Vietnam

## Abstract

**Background:**

To evaluate stage changes when applying the 9th edition of the TNM classification instead of the 8th edition in Vietnamese patients with nasopharyngeal carcinoma.

**Method:**

A cross-sectional descriptive study was conducted in newly diagnosed patients with nasopharyngeal carcinoma treated at the Department of Head and Neck Radiation Oncology of Vietnam National Cancer Hospital (K Hospital) from January 2025 to December 2025.

**Results:**

The study included 555 patients, with a mean age of 51.8 ± 13.5 years. The male-to-female ratio was 2.53/1. Most patients had non-keratinizing squamous cell carcinoma, undifferentiated subtype, accounting for 99.3%. According to the 8th edition of the TNM classification, the distributions of T stage, N stage were 25,4% T1, 25,9% T2, 23,4% T3, 24,3% T4; 13,3% N0, 37,8% N1, 33,0% N2, 15,9% N3; and Stage III-IVA accounting for 64.1%. When reassessed using the 9th edition, there were 6 patients experienced changes in N stage, and 88.3% of patients changed overall stage. This reclassification affected treatment decisions, with 1 patient having their standard treatment approach modified based on the 9th edition of the TNM classification.

**Conclusion:**

The 9th edition of the TNM classification introduces several changes compared with the 8th edition, potentially improving prognostic assessment and patient stratification.

## 1 Introduction

Nasopharyngeal carcinoma (NPC) is a common head and neck malignancy, with a high prevalence in East and Southeast Asia (1). According to GLOBOCAN 2022, there were 122,434 new cases and 73,428 deaths related to NPC (2). Vietnam is located in close geographical proximity to Southern China, particularly Guangdong and Guangxi provinces, which are recognized endemic regions for nasopharyngeal carcinoma (NPC). Consistent with this epidemiological background, NPC represents a significant cancer burden in Vietnam, ranking ninth in incidence and eighth in cancer-related mortality nationwide. As the largest oncology center in the country and the major referral hospital in Northern Vietnam, National Cancer Hospital (K Hospital) treats thousands of NPC patients annually. The hospital also plays a central role in the national oncology network, providing professional guidance to provincial hospitals and leading the implementation of updated staging systems and modern treatment protocols for cancer management across Vietnam. The AJCC/UICC TNM staging system is the most widely used classification system worldwide (3). The 8th edition of the TNM staging system, released in 2017, has been widely used for many years and has played a major role in both clinical management and research. However, several limitations have been identified, including suboptimal prognostic stratification for cases with limited bone invasion, insufficient risk assessment for extranodal extension, and the inability to differentiate patients with metastatic disease into distinct prognostic subgroups (4). In response to these limitations, the 9th edition of the TNM staging system for NPC was developed and published in January 2025, introducing significant changes in prognostic stratification and treatment guidance (5). In current clinical practice, several major cancer centers worldwide have gradually begun to adopt this new TNM staging system. However, in Vietnam, no studies have yet evaluated the application of the 9th edition TNM classification, particularly regarding changes in stage distribution and treatment orientation compared with the 8th edition. Therefore, this study was conducted to assess changes in stage classification among real-world patients diagnosed with NPC when using the TNM 9th edition instead of the TNM 8th edition.

## 2 Materials and methods

### 2.1 Patients

555 newly diagnosed patients with histologically confirmed NPC were treated at the Department of Head and Neck Radiation Oncology, K Hospital, from January 2025 to December 2025, meeting the following criteria:

#### 2.1.1 Inclusion criteria

- Patients with a confirmed diagnosis of nasopharyngeal carcinoma (NPC)
- Lesions evaluated using imaging modalities, including contrast-enhanced head and neck MRI and whole-body CT
- Complete and available follow-up records

#### 2.1.2 Exclusion criteria

- Patients with a previous history of NPC
- Incomplete or unavailable medical records

### 2.2 Research location

Department of Head and Neck Radiation Oncology, K Hospital

### 2.3 Resarch methods

*Study design:* Cross-sectional descriptive study
*Sample size:* Convenience sampling, including 555 newly diagnosed patients with NPC treated at the Department of Head and Neck Radiation Oncology, K Hospital.

### 2.4 Research process

- Create a list of newly diagnosed NPC cases from January 2025 to December 2025.
- Collect medical records according to the research medical record template.
- Collect research variables, including:
  + Clinical characteristics: age, gender
  + Histopathological type
  + Imaging characteristics of the tumor and lymph nodes on CT and MRI
  + Characteristics of metastatic lesions (if any)
  + Treatment regimen

### 2.5 Data analysis

Collected data were coded and analyzed using SPSS version 26. Variables were summarized using means, medians, and proportions, and differences between proportions were assessed using the chi-square test.

### 2.6 Research ethics

Descriptive research, non-interventional, aimed at improving the quality of treatment. Data is truthful and objective.

## 3 Results

### 3.1 Baseline characteristics

The characteristics of the study population are summarized in **Table 1**. The mean age of the patients was 51.8 ± 13.5 years, ranging from 10 to 87 years. The male-to-female ratio was 2.53:1. The most common histopathological type was non-keratinizing squamous cell carcinoma, undifferentiated subtype, accounting for 99.3% of cases. According to the TNM 8th edition, the proportions of T1, T2, T3, and T4 tumors were 25.4%, 25.9%, 23.4%, and 24.3%, respectively; the proportions of N0, N1, N2, and N3 disease were 13.3%, 37.8%, 33.0%, and 15.9%, respectively. Advanced-stage disease (stage III–IVA) predominated, accounting for 64.1% of cases, followed by stage II at 22.2%.

**Table 1:** Baseline characteristics.

Characteristics		N	%
Age group	≤40 years	127	22.9
	40–60 years	264	47.6
	>60 years	164	29.5
Mean age	51.78 ± 13.47 (10-87)		
Sex	Male	398	71.7
	Female	157	28.3
Histopathology	Keratinizing squamous cell carcinoma	4	0.7
	Non-keratinizing squamous cell carcinoma, undifferentiated subtype	551	99.3
	Basal cell carcinoma	0	
T stage according to TNM 8	T0	5	0.9
	T1	141	25.4
	T2	144	25.9
	T3	130	23.4
	T4	135	24.3
N stage according to TNM 8	N0	74	13.3
	N1	210	37.8
	N2	183	33.0
	N3	88	15.9
M stage according to TNM 8	M0	508	91.5
	M1	47	8.5
Stage according to TNM 8	I	29	5.2
	II	123	22.2
	III	180	32.4
	IVA	176	31.7
	IVB	47	8.5
Treatment according to TNM 8	Radiotherapy alone (RT)	41	7.4
	Concurrent chemoradiotherapy (CRT)	126	22.7
	Induction chemotherapy (IC) → Concurrent chemoradiotherapy (CRT)	341	61.4
	Palliative chemotherapy	47	8.5

### 3.2 Application of the TNM 9^th^ edition

When classified according to the TNM 9th edition, the proportions of N0, N1, N2, and N3 disease were 13.3%, 37.5%, 32.3%, and 16.9%, respectively. Overall stage distribution also changed, with stages II and III accounting for the majority of cases (64.5%) (**Table 2**).

**Table 2:** Stage classification according to the TNM 9th edition.

Characteristics		N	%
T stage	T0	5	0.9
	T1	141	25.4
	T2	145	26.1
	T3	130	23.4
	T4	134	24.1
N stage	N0	74	13.3
	N1	208	37.5
	N2	179	32.3
	N3	94	16.9
M stage	M0	508	91.5
	M1A	15	2.7
	M1B	32	5.8
Stage	IA	41	7.4
	IB	109	19.6
	II	178	32.1
	III	180	32.4
	IVA	15	2.7
	IVB	32	5.8

When applying the TNM 9th edition to cervical lymph node assessment, two cases were upstaged from N1 to N3 and four cases from N2 to N3, accounting for 1.1% (**Table 3**). In terms of overall stage grouping, 490 patients experienced stage changes, corresponding to 88.3% (**Table 4**).

**Table 3:** Changes in N-stage when applying the TNM 9th edition compared with the TNM 8th edition.

		N stage – TNM 9th				Total
		0	1	2	3	
N stage – TMN 8^th^	**0**	**74**	0	0	0	**74**
	**1**	0	**208**	0	2	**210**
	**2**	0	0	**179**	4	**183**
	**3**	0	0	0	**88**	**88**
Total		**74**	**208**	**179**	**94**	**555**

Bold: case that no change when using TNM9 rather than TNM8.

**Table 4:** Changes in stage when applying the TNM 9th edition compared with the TNM 8th edition.

		Stage – TNM 9th						Total
		IA	IB	II	III	IVA	IVB	
Stage TNM 8^th^	**I**	**29**	0	0	0	0	0	**29**
	**II**	12	109	**1**	1	0	0	**123**
	**III**	0	0	177	**3**	0	0	**180**
	**IVA**	0	0	0	176	**0**	0	**176**
	**IVB**	0	0	0	0	15	**32**	**47**
Total		**41**	**109**	**178**	**180**	**15**	**32**	**555**

Bold: case that no change when using TNM9 rather than TNM8.

When applying the TNM 9th edition, the standard treatment strategy changed in one case (0.2%), from concurrent chemoradiotherapy to induction chemotherapy (**Table 5**).

**Table 5:** Changes in standard treatment strategies.

		Treatment – TNM 9th				Total
		RT	CRT	IC	Pallative ChT	
Treatment TNM 8th	**RT alone**	**41**	0	0	0	**41**
	**CRT**	0	**125**	1	0	**126**
	**IC**	0	0	**341**	0	**341**
	**Pallative ChT**	0	0	0	**47**	**47**
Total		**41**	**125**	**342**	**47**	**555**

Bold: case that no change when using TNM9 rather than TNM8.

## 4 Discussion

NPC is a common head and neck malignancy with distinct epidemiological characteristics. In endemic regions, the age distribution shows a bimodal pattern, with one peak in the 15–24-year age group and a second peak in the 65–79-year age group (1). NPC occurs more frequently in males than in females, with a reported male-to-female ratio ranging from 2.0 to 2.5. In our study, the mean age was 51.8 ± 13.5 years, with a male-to-female ratio of 2.53:1, and the most commonly affected age group was 40–60 years, accounting for 47.6% of cases. Similar results have been reported in other studies; Du et al. (4) reported a mean age of 45.4 years (4); Chen et al. (6), in a study of 5130 patients with NPC in China, reported that the most common age group was 40–49 years, accounting for 36% of cases, and that males predominated, comprising 73.4% of patients (6).

In NPC, non-keratinizing squamous cell carcinoma of the undifferentiated subtype is the most common histopathological type, accounting for more than 95% of cases in endemic regions (7). In our study, this subtype represented 99.3% of cases. Similarly, Du et al. reported 2970 cases of non-keratinizing undifferentiated squamous cell carcinoma, comprising 99.3% of their cohort, consistent with our findings (4).

When staging NPC according to the TNM 8th edition, among the 555 patients in our study, the proportions of T1, T2, T3, and T4 disease were 25.4%, 25.9%, 23.4%, and 24.3%, respectively. Other studies worldwide using the TNM 8th edition have reported different distributions. Pan et al., in a study of 4701 patients with non-metastatic NPC, found that T3 disease was the most prevalent, accounting for 44.3% of cases (5). Du et al. reported T-stage proportions of 3.8%, 15.4%, 46.5%, and 34.2%, respectively (4). These discrepancies may be explained by differences in sample size and endemic regions. Regarding nodal staging, the majority of patients in our cohort had lymph node metastases, accounting for 86.7%, predominantly at N1 and N2 stages. Similarly, in Du’s study, 2591 of 2990 patients had clinically and radiologically confirmed nodal metastases, with 35.8% classified as N2–N3 disease (4). Distant metastases were present at diagnosis in 47 patients, corresponding to a rate of 8.5%, which is higher than that reported by Pan et al., in whom distant metastases accounted for 4.33% of cases (5).

The treatment of NPC is multimodal. In our study, 84.1% of patients received concurrent chemoradiotherapy (with or without induction chemotherapy), 7.4% were treated with radiotherapy alone, and 8.5% received palliative chemotherapy due to distant metastatic disease. These results are largely consistent with reports from other studies worldwide, Du et al. reported an overall rate of concurrent chemoradiotherapy of 95.8%, with 4.2% of patients receiving radiotherapy alone (4); Another study reported rates of radiotherapy alone and concurrent chemoradiotherapy of 16.75% and 83.25%, respectively (8).

When applying the new TNM 9th edition, the T classification remained unchanged; however, several factors discussed in previous studies—particularly those from Chinese cohorts—have been shown to influence patient prognosis. First, pterygoid muscle invasion was downstaged from T4 in the TNM 7th edition to T2 in the TNM 8th edition. When comparing prognosis between patients with and without pterygoid muscle invasion, Du et al. reported no significant differences in 5-year overall survival (90.9% vs. 91.5%, p = 0.972) or distant metastasis–free survival (90.9% vs. 89.1%, p = 0.787). In contrast, Pan et al. reported that invasion of the medial or lateral pterygoid muscle was associated with worse overall survival compared with no invasion (adjusted hazard ratio [aHR] 2.52; 95% CI, 1.48–4.26) (4, 5). Owing to these conflicting findings and the minimal impact on treatment strategies, upstaging to T3 in the TNM 9th edition was not recommended by the AJCC and UICC committees (only 3 of 12 members supported this change) (5). Second, attention has been drawn to the subgroup of patients with early clival invasion, defined as tumor invasion limited to the pterygoid process and/or the sphenoid bone. Li et al. demonstrated that patients with T3 disease and early clival invasion had a better prognosis than those with extensive T3 invasion, with respect to overall survival (91.9% vs. 88.8%, p = 0.025), failure-free survival (83.9% vs. 78.5%, p = 0.007), and distant metastasis–free survival (90.5% vs. 86.3%, p = 0.012) (4, 9, 10). Moreover, these studies showed no significant survival difference between patients with early T3 clival invasion and those with T2 disease. Several previous studies have also suggested that this early T3 subgroup does not derive benefit from induction chemotherapy (10). Based on these findings, some authors have proposed revising the T classification according to the extent of bone invasion. However, this proposal has not reached consensus, as other validation studies did not support such conclusions (5). Larger, contemporary studies may therefore be required, particularly in the era of advanced imaging, to more accurately assess bone invasion, refine prognostic stratification, and potentially inform future revisions of the T classification.

**Figure 1:**
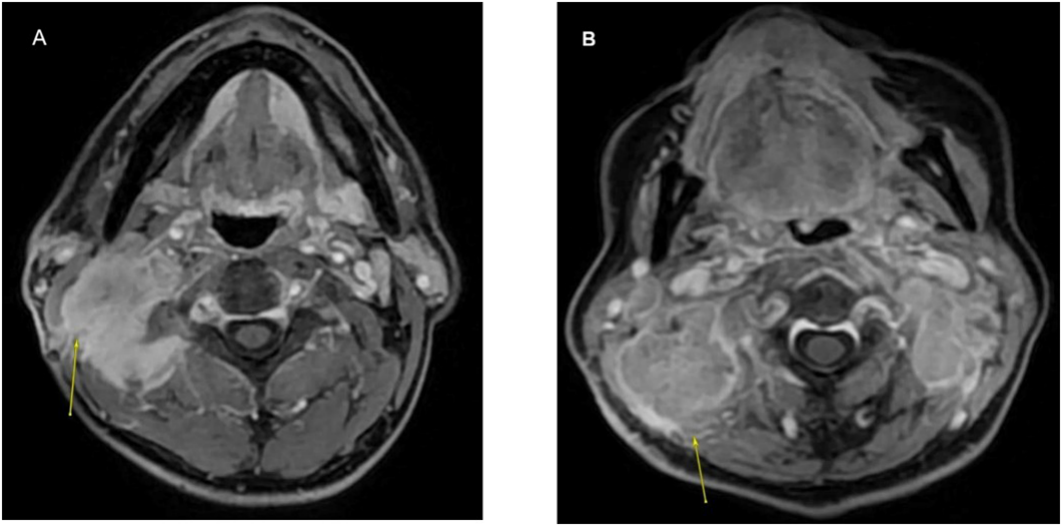
Illustration of nodal stage changes. Reclassification from N1 to N3 **(A)** and from N2 to N3 **(B)** according to the updated staging system.

The N classification in the TNM 9th edition shows a marked change for patients with extranodal extension (ENE) (**Figure 1**). Imaging features of ENE are graded as grade 0 (no extranodal extension), grade 1 (capsular disruption with invasion into surrounding fat), grade 2 (capsular disruption with matted lymph nodes), and grade 3 (capsular disruption with invasion of adjacent structures) (4, 5, 11). Studies have demonstrated that patients with G3 ENE have a significantly worse prognosis and a higher risk of distant metastasis (4, 5, 11, 12). Lu et al. (12), in a study of 1390 patients with NPC and nodal metastases, reported 487 patients with G2 ENE and 83 with G3 ENE. Compared with patients with G0/1 ENE, those with G2/3 ENE had an increased risk of distant metastasis (HR 2.05 and 3.18, respectively; p < 0.001) and a higher risk of mortality (HR 1.62 and 2.30; p = 0.002 and p < 0.001, respectively) (12). A multicenter study by Du et al. reported similar findings, showing that patients with N1–N2 disease and G3 ENE had lower 5-year overall survival than those with N1–N2 disease without G3 ENE (82.0% and 77.1% vs. 90.7% and 87.0%, respectively), with survival comparable to that of N3 disease in the TNM 8th edition (78.7%; log-rank p = 0.626 and 0.976) (4). Based on this evidence, upstaging lymph nodes with extranodal extension invading adjacent structures to N3 was recommended in the TNM 9th edition by the AJCC and UICC committees (12/12 members in agreement) (5). In our study, two patients were upstaged from N1 to N3 and four from N2 to N3. Multiple imaging modalities are used to assess extranodal extension, with CT and MRI being the most commonly employed; however, MRI is generally preferred because of its superior soft-tissue contrast provided by multiple sequences, including fat-suppressed T2-weighted and post-contrast T1-weighted images (13). This observation is consistent with our findings, as all six patients with N-stage changes due to extranodal extension were identified based on head and neck MRI.

The M classification also underwent a fundamental change in the TNM 9th edition. The M1 category was subdivided into M1A and M1B based on the number of metastatic lesions. This modification was supported by several studies evaluating patients with metastatic NPC, which demonstrated a statistically significant difference in 5-year overall survival between patients with three or fewer metastatic lesions and those with more than three lesions (59.1% vs. 29.2%, p < 0.001 in Du et al.; 60.7% vs. 44.2%, p = 0.01 in Pan et al.) (4, 5). Applying this revised classification in our cohort, among 47 patients with distant metastases, 15 were classified as M1A and 32 as M1B. This stratification facilitates more appropriate treatment selection. In particular, consolidative radiotherapy following chemotherapy has been investigated by multiple authors and has been shown to significantly improve overall survival. Zhou et al., in a study of 977 patients with metastatic NPC at Sun Yat-sen University Cancer Center, further stratified metastatic disease into M1A (single-site metastasis without liver involvement), M1B (multiple-site metastases without liver involvement), and M1C (liver metastasis). The reported 3-year overall survival rates ranged from 54.5% to 72.8%, 34.3% to 41.6%, and 22.6% to 23.6%, respectively (14). Consolidative radiotherapy was primarily indicated for patients with single-site metastasis and no liver involvement, with a marked improvement in survival outcomes.

The TNM 9th edition has led to substantial changes in overall stage grouping. Specifically, patients with T1–2N0M0 disease are classified as stage IA, while those with T1–2N1 disease are assigned to stage IB. Patients previously classified as stage III and IVA under the TNM 8th edition are downstaged to stages II and III, respectively. In addition, patients with M1A disease are classified as stage IVA, whereas those with M1B disease are classified as stage IVB. The redefinition of stages IA and IB is supported by the study of Du et al., which demonstrated that the 5-year overall survival of patients with T2N0 disease (98.6%) was significantly higher than that of patients with T1–2N1 disease (94.7%, p = 0.044) and was comparable to that of patients with T1N0 disease (4). In our study, 490 of 555 patients experienced changes in overall stage grouping, with a shift from predominantly stage III and IVA disease (64.1%) under the TNM 8th edition to predominantly stage II and III disease (64.5%) under the TNM 9th edition. This distribution is consistent with the epidemiology of NPC in Vietnam, where most patients are diagnosed with locally advanced disease. Overall, the application of the TNM 9th edition stage grouping provides improved prognostic stratification and more accurate prediction of treatment outcomes.

Because changes in stage classification mainly involved the N category, treatment strategies were also affected. In our study, when applying the TNM 9th edition, only one patient (0.2%) experienced a change in the standard treatment approach (excluding other factors such as comorbidities and performance status). Specifically, this patient was shifted from concurrent chemoradiotherapy to induction chemotherapy due to upstaging from N1 to N3. For N3 disease, an induction chemotherapy followed by concurrent chemoradiotherapy regimen is preferred, as it has demonstrated efficacy in both locoregional control and systemic disease control (15). This change can clearly influence treatment outcomes. However, the proportion of patients with altered treatment strategies was relatively low compared with the extent of changes in overall stage grouping. This is partly because, in the present study, treatment decisions were based on existing NCCN and ESMO guidelines derived from the TNM 8th edition, as updated recommendations aligned with the TNM 9th edition are not yet available. In the future, emerging evidence—particularly from studies conducted in China—suggests a trend toward treatment de-escalation, including reduced use of chemotherapy combinations as well as lower radiation doses and target volumes, with preliminary results indicating outcomes comparable to standard treatment. Consequently, it is likely that international oncology organizations will issue updated recommendations for treatment selection in patients with NPC.

## 5 Conclusion

Since January 2025, the AJCC/UICC has introduced the TNM 9th edition for nasopharyngeal carcinoma. Our preliminary findings show that this updated system leads to meaningful changes in staging, prognostic stratification, and treatment guidance. Further implementation and validation studies are needed to confirm its clinical value and inform future refinements of the TNM classification.

## Data Availability

The raw data supporting the conclusions of this article will be made available by the authors, without undue reservation.
